# Computational and systems neuroscience: The next 20 years

**DOI:** 10.1371/journal.pbio.3002306

**Published:** 2023-09-26

**Authors:** Christopher Summerfield, Kevin Miller

**Affiliations:** 1 Google DeepMind, London, United Kingdom; 2 Department of Experimental Psychology, University of Oxford, Oxford, United Kingdom; 3 Department of Ophthalmology, University College London, London, United Kingdom

## Abstract

What does the future of neuroscience look like in the age of big neural data and advanced AI models. In this Perspective, the authors advocate for new approaches that combine the merits of data-driven model discovery and hypothesis-driven model comparison.

## Teaser

Over the past 20 years, neuroscience has been propelled forward by theory-driven experimentation. We consider the future outlook for the field in the age of big neural data and powerful artificial intelligence models.

This article is part of the *PLOS Biology* 20th Anniversary Collection.

*PLOS Biology* was first published in 2003. That year, important changes were afoot in the field of neuroscience. The marriage of neural recording with computational theory was just starting to bear serious fruit, prompting the inauguration of the Computational and Systems Neuroscience (Cosyne) meeting in 2004. In tandem, three research streams were attracting particular interest. First, researchers studying the primate oculomotor system had just shown that neural activity accompanying a decision to move the eyes bore many of the hallmarks of venerable models of decision latencies, including noisy accumulation-to-bound dynamics that approximate Bayesian inference [1] and recurrent inhibition in neural networks [2]. Second, both single-neuron electrophysiology and functional MRI were being deployed to study the motivational signals in basal ganglia and medial prefrontal cortex that accompany reward-guided decisions, building on theoretical constructs from the fields of Reinforcement Learning (RL) [3] and behavioral economics [4] (the Society for Neuroeconomics also held its first annual meeting in 2003). Third, a team of researchers at Cold Spring Harbor demonstrated the viability of rodent models for studying decision-making [5], complementing burgeoning research into spatial memory and navigation in rats; grid cells would be discovered just 2 years later [6].

In the 20 years since, the seeds laid by these (and many other) research strands have grown strong. Modeling frameworks based on psychophysics, Bayesian methods, connectionist networks, RL, and econometric models have been extensively used to test mechanistic theories and explain neural data. In parallel, more overtly neurally grounded models have sprung up, such as those that account for a cornucopia of cell types supporting navigation and memory in rodents, the discovery of which earned a Nobel Prize in 2014. More generally, over the past 20 years, research linking models and data has become the norm, pointing the field towards a golden era of genuinely cumulative science. Today, we have inherited sophisticated theories of core brain functions, including (but not limited to) sensorimotor choice, reward-guided learning, visual attention, memory, and navigation.

The first published issue of *PLOS Biology* presciently drew attention to another theme that was to define 21st century neuroscience. An essay in its very first edition, entitled “Neuroscience Networks: Data-sharing in an Information Age” [7], included the following quote: “As we emerge from the ‘decade of the brain,’ we are entering a decade for which data-sharing will be the currency for progress in neuroscience.” How right they were! In the time since, dramatic advances in high-throughput experimental methods have resulted in vast datasets of behavior, brain anatomy and connectivity, and neural recordings at a level of scale and detail that would have been unthinkable 20 years ago [8]. However, perhaps disappointingly, this era of “neural big data” has not automatically furnished transformative new insights about how the brain works. Rather, it has triggered growing debate about whether the classical theoretical frameworks that have sustained us for the past 20 years will continue to prove adequate or whether entirely new classes of computational approach are needed to make sense of this glut of data.

The parameters of the debate have been set by fast-paced developments in the sister field of artificial intelligence (AI) research. These have impacted neuroscience in 2 major ways. First, the arrival of deep learning systems that exhibit naturalistic competences rivaling those of humans (in object recognition, expert game play, and natural language) has dramatically revived the question (first posed in the 1980s connectionist movement) of whether AI systems can be deployed as plausible process models of perception and cognition [9]. Can deep neural networks step into the role that has been held so ably by classical modeling frameworks (chronometric, econometric, Bayesian, RL) and be taken seriously as computational simulacra of brain processes in humans and other animals? Enthusiasm about deep learning models has been driven by studies showing linearly related neural activity in biological and artificial systems performing comparable tasks, such as image labeling or sentence completion [10]. However, because these models are trained to perform the task (rather than fit the data), this approach does not naturally lend itself to cumulative science through a careful cycle of experimentation and model refinement because any correspondence between computation in vivo and in silico is inevitably serendipitous. In other words, when we find that brains only partially mimic AI tools (or vice versa), it is not always clear how to adjust the models to behave as more plausible biological theories. Another issue looming over these approaches is that, despite our ready access to their parameters and dynamics, interpreting these models presents a formidable challenge. This work has provoked a spectrum of reactions, from calls to retreat back to stylized models with a handful of free parameters, to triumphalist claims that neuroscience should give up on explanations altogether, and embrace million-parameter deep learning models as “the answer” to whatever question neuroscientists were trying to address in the first place.

The second prong of AI research to impact neuroscience is the development of methods for discovering interpretable latent structure in high-dimensional data. These methods can be readily applied to newly available large-scale neuroscience datasets, including both spontaneous behavior and neural recordings [11]. So far, however, these methods have mainly been deployed for summarizing and visualizing complex datasets, making only limited contact with established theories that form the bedrock of our understanding of cognition and behavior. One promising path forward is the development of tools that combine elements of explainable classic models with flexible AI systems, affording the expressiveness to fit complex datasets while retaining the merits of interpretability and hypothesis-driven science. For example, researchers can fit neural networks directly to behavior data and then assess the relative consequences of tightening or relaxing various assumptions [12], up to the point where a full interpolation between classic and deep learning models is available [13]. Another recent approach is to add structural constraints to recurrent neural networks to encourage them to learn cognitively interpretable solutions [14]. We hope that this will ultimately furnish a set of tools that can liberate neuroscience from a reliance on the overly simple handcrafted models of yesteryear, allowing discovery of new theories directly from our datasets, but without compromising the commitment to hypothesis-driven science that has sustained our field for the past 2 decades.

Over the next 20 years of neuroscience, it will be critical to maintain clear goals for our field. Tools from AI research offer an Aladdin’s Cave of new modeling opportunities for neuroscientists. However, they also open a Pandora’s Box of potential confusion about the boundaries between neuroscience and adjacent disciplines more focused on solving engineering problems than understanding biology. Neuroscience is a natural science whose ultimate goal—to deliver a human-interpretable understanding of the inner workings of the mind and brain—remains as urgent as ever, from both curiosity-driven and translational standpoints. AI systems will have an important role, both as theories in themselves and as tools for discovering new hypotheses. However, using them effectively will require navigating the theoretical minefields outlined above: finding ways to harness very large datasets and very large models while maintaining a commitment to cumulative hypothesis-driven science and interpretable theories of brain function.
